# Intentions to leave the job and nursing profession among nurses in Kenya’s referral hospitals: exploring the effects of organizational culture, work-related stress and job satisfaction

**DOI:** 10.1186/s12912-025-03422-0

**Published:** 2025-07-02

**Authors:** Evans Kasmai Kiptulon, Miklós Zrínyi, Adrienn Ujváriné Siket

**Affiliations:** 1 Doctoral School of Health Sciences, Faculty of Health Sciences, University of Pécs, Pécs, Hungary; 2 Kenya Medical Training College- Kapenguria Campus, Po Box 576-30600, Kapenguria, Kenya; 3 The County Government of West Pokot, Ministry of Health, Po Box 222-30600, Kapenguria, Kenya; 4 Doctoral School of Health Sciences, Faculty of Health Sciences, University of Debrecen, Debrecen, Hungary

**Keywords:** Intention to leave career, Job satisfaction, Job turnover intention, Kenya, Organizational culture, Work-Related stress, Profession turnover intention

## Abstract

**Background:**

Kenya’s healthcare system, like many in Low- and Middle-Income Countries, is grappling with a severe shortage of nurses, yet experiences continuous mass exodus and high turnover. While understanding, and early suppression of nurses’ job and professional turnover intentions is crucial for tackling this crisis, there are limited studies conducted in Kenya focusing on major predictors of nurse turnover intentions, including organizational culture, work-related stress and job satisfaction. This study therefore aimed to fill this gap.

**Aim:**

To explore the effects of organizational culture, work-related stress and job satisfaction on nurses’ job and professional turnover intentions in Kenya.

**Methods:**

This cross-sectional study surveyed 429 nurses in Kenya’s major referral hospitals. Validated tools were used to assess organizational culture (OC), Work-Related Stress (WRS), job satisfaction (JS), job Turnover Intention (TI) and Profession Turnover Intention (ProfTI). Data were analysed using SPSS v28 with Chi-square, correlation and logistic regression at a 95% confidence level(P < .005).

**Results:**

Our analysis revealed that 81.4% and 31.4% of Kenyan nurses intend to leave their jobs and the nursing profession respectively. The overall organizational culture was largely neutral with a slight inclination to positive (M = 2.54, SD = 0.62), work-related stress was moderately high (M = 2.92, SD = 0.51), and job satisfaction was low, with only 56.6% of nurses reporting satisfaction. Predictors of TI were Age (OR = 0.45, 95% CI [0.26,0.078], P = .004), years of work experience (OR = 0.40, 95% CI [0.17, 0.93], P = .033) and job satisfaction (OR = 0.45,95% CI [0.26, 0.77], P = .004). Contrary to many existing literature, WRS inversely predicted TI (OR = 0.63, 95% CI [0.40, 0.99], P = .046). Predictors of ProfTI were: marital status(OR = 3.19, 95% CI [1.70,5.99], P = .001), working in surgical wards (OR = 12.70, 95% CL [1.48,108.85], P = .020) or in ICU/renal/theatre (OR = 10.79, 95% CI [1.27, 91.45] P = .029), salary(OR = 4.91,95% CI[1.21,19.92], P = .026),Job satisfaction (OR = 35, 95% CI [0.23, 0.54], P = < 0.001) and WRS (OR = 1.74, 95%, CI [1.15. 2.61], P = .008). Organizational culture did not significantly predict TI or ProfTI.

**Conclusion:**

Kenya’s major referral hospitals are facing a crisis, marked by high rates of both TI and ProfTI. Urgent action is required from hospital managers and administrators, policymakers, the Kenyan government, and all other key stakeholders to enhance job satisfaction, reduce work-related stress, foster a strong positive organizational culture, and improve salaries.

**Clinical trial number:**

Not applicable.

**Supplementary Information:**

The online version contains supplementary material available at 10.1186/s12912-025-03422-0.

## Background

The global healthcare systems, especially in Low- and Middle-Income Countries (LMICs) including Kenya, are grappling with a severe shortage of nursing personnel due to high nurse turnover and profession exit. This crisis continues to escalate, threatening the stability and quality of healthcare delivery in these fragile economies [1–5]. According to the World Health Organization (W.H.O), the world faced a shortfall of 7.07 million nurses in 2020, with 89% of this deficit concentrated in LMICs. Projections indicate that by 2035, this shortfall will double and could further triple by 2050 if immediate action is not taken to address turnover and profession leaving among nurses [2, 6, 7].

Nurses intention to leave their jobs, also referred to as Turnover Intention (TI), is the desire of a nurse to leave their current job and employer within a specific timeframe [8]. TI serves as the primary predictor of actual turnover, which refers to a complete departure, termination or exit of nurses from their current employment [8–10]. On the other hand, Profession Turnover Intention (ProfTI) or Intention to Leave Profession(ITLProf) refers to the nurse’s expressed desire or plan to permanently exit the nursing profession altogether rather than merely changing employers or positions [11]. ProfTI represents a more severe form of occupational withdrawal, often driven by chronic dissatisfaction, burnout, poor work-environments, or lack of professional growth and general poor management of Human Resources for Health(HRH) [12–14].

Turnover and professional exits are healthcare crisis causing workforce instability, increased workloads for the remaining nurses, and crippling costs to healthcare facilities [15, 16]. Other negative consequences of turnover and professional leaving include poor quality of patient care, staff shortages, increased medical errors, longer waiting times, and higher patient readmission rates [16, 17]. With increased turnover and professional leaving, the remaining nurses are overburdened and face high burnout, further fueling the cycle of turnover and career leaving [12, 18]. Hospitals also struggle with disrupted care continuity, loss of skilled nursing experts who are hard to replace, weakened mentorship, and dealing with service quality, especially in underserved areas. The crisis of turnover and nurses leaving the profession in LMIC countries like Kenya, which already have a huge shortage of nurses threatens to completely collapse the healthcare system if left unaddressed [1, 19].

Nurse turnover and nurses leaving the profession has emerged as a critical nursing and public health problem in Kenya [5, 20]. Kenya, a country with a population of 56 million people, had a total of 189,932 healthcare workers in 2020, of whom 58% were nurses [21]. Despite this, the healthcare worker-to-patient ratio stood low at 13.8 per 10,000, significantly lower than the WHO-recommended 44.5 per 10,000 [22, 23]. In addition to this crisis, Kenya’s nurse density declined from 128.3 in 2018 to 112 per 100,000 population in 2022 far below the WHO-recommended of at least 220 nurses per 100,000 people for developing countries [23, 24]. This ongoing decline is driven by persistent turnover and the “brain drain” phenomenon, whereby Kenyan nurses leave the country for better opportunities abroad or completely exit the profession for other non-nursing professions. According to the Nursing Council of Kenya (NCK), an average of 840 nurses apply for certificate verification annually intending to migrate overseas [25, 26]. The gravity of this issue was amplified in April 2025, when *Daily Nation*, Kenya’s leading newspaper, reported a mass exodus of nurses—highlighting a staggering 1,000—staff gap at Moi Teaching and Referral Hospital (Kenya’s second major referral hospital) as more nurses leave for jobs abroad [27].

## Organizational culture, work-related stress and job satisfaction

Amidst the pressing problem of turnover and profession leaving, understanding TI and ProfTI, together with the concepts of Organizational Culture(OC), Work-Related Stress(WRS) and Job Satisfaction(JS) emerge as pivots and the major lenses through which this problem can be better understood and managed [28–30], yet remains understudied and poorly documented in Kenya. Organizational culture is defined as a system of shared values, beliefs and morals that produces norms of behaviour and establishes an organizational way of life [31], or simply “how do we do things around and within hospitals” [32], is a powerful variable in nursing work environments influencing work-related stress, job satisfaction and eventually TI and ProfTI. A strong positive organizational culture characterized by respect for nurses, teamwork, collaboration, nurses’ involvement in leadership and decision making, effective communication, flexible work schedules, adequate resources and staffing, high opportunities for career and academic progression, adequate pay, regular recognition, good leadership styles, and with decent work environments enhances job satisfaction ultimately reducing TI and ProfTI [29, 33–41]. Conversely, a negative, weak or toxic culture breeds dissatisfaction, increases work-related stress, and fuels employees’ desire to leave [30, 38, 42–49].

Despite the importance of OC, WRS and JS roles in TI and ProfTI among nurses, there is an inadequacy of dedicated studies in Kenya. Most global studies done on the impact of OC, WRS and JS on TI and ProfTI have focused mainly on high-income countries [29, 30, 43, 50–53]. A systematic review conducted in Sub-Sahara Africa(SSA) [1], though lacking Kenya-specific data, found that in SSA, the pooled prevalence of nurses TI stood at 50.74%, with East Africa sub-region—where Kenya is a member—reporting the highest proportion(58.03%), compared to the lowest in Southern Africa region(33.04%). Although Kenya is facing severe nursing shortage and escalating rates of both job and profession leaving, there remains a critical gap in understanding TI and ProfTI—the primary predictors of (job and profession leaving), and their main influencing factors such as OC, WRS and JS. This insufficiency of dedicated studies has led to a lack of targeted solutions to mitigate nurses leaving both their jobs and the nursing profession in Kenya. This study therefore aims to fill this gap.

## Research objectives

i. To characterize the prevailing organizational culture of Kenya’s major referral hospitals as viewed by nurses.
ii. Determine the level of work-related stress among nurses working in Kenya’s major referral hospitals.
iii. To determine the prevalence of job and profession turnover intention among nurses working in Kenya’s major referral hospitals.
iv. To determine the level of job satisfaction among nurses working in Kenya’s major referral hospitals.
v. To examine the predictive influence of organizational culture, work-related stress, and job satisfaction on the job and profession turnover intentions among nurses working in Kenya’s major referral hospitals.

## Methodology

### Research design

This study utilized a cross-sectional design. This design was deemed appropriate as it involved collecting data at a single point in time without the need for prolonged participant follow-up.

### Study area

This study was conducted in fourteen major referral hospitals across Kenya, including twelve government-run and two private referral facilities. A referral hospital is a medical facility that serves as a center for specialized medical care, research, and education within a country that receives patients from lower-level health facilities such as dispensaries, health centers and sub-county hospitals. Typically, it provides an advanced and specialized range of medical services, including diagnostic, surgery, paediatrics, obstetrics, general medicine, and gynecology services and typically has specialists and consultants. Kenya, a middle-income country in East Africa, has an extensive healthcare system comprising approximately 9,696 facilities according to the data from the country’s Ministry of Health [54]. These facilities are organized in a hierarchical structure, from national referral hospitals at the top, followed by county and sub-county hospitals, health centres, dispensaries and community health units at the lower levels. There are eight national referral Hospitals—a mix of both government-owned and privately operated facilities— and 47 County referral hospitals, one in each of the 47 counties. Nurses working in these institutions hold diverse academic qualifications, ranging from certificate in nursing, diploma in nursing, and Bachelor of Science in Nursing (BScN) to Doctor of Philosophy (PhD).

### The target and the study population

The target population included all nurses in Kenya, while the study population consisted of nurses working in selected major national, county, and private referral hospitals. As of 2024, the Nursing Council of Kenya estimates the country’s nursing workforce at approximately 81,564 nurses [55].

### The sample size determination

Using Fisher’s formula for prevalence studies [56], and assuming a 95% confidence level, a prevalence (P) of 0.5, and a 5% margin of error, the calculated sample size was 384. After adjusting for a 10% non-response rate, the final sample size was set at 423.

n = desired sample size (if the target population is greater than 10,000) Where: Z = std normal deviate at the desired 95% confidence level (1.96), P = prevalence. Q = 1-P, D = degree of accuracy desired, here set at 0.05.

### Sampling technique and procedure

A multistage sampling technique was employed to select participants. In the first stage, five national referral hospitals were randomly chosen through a lottery selection from a list of eight national referral hospitals currently in Kenya. Two major private referral hospitals were also selected. For county referral hospitals, Kenya was subdivided into eight former provincial regions of Central, Coast, Eastern, Nairobi, Northeastern, Nyanza, Rift Valley and Western and a box was created representing each region. Counties within each provincial unit, along with their referral hospital, were written on pieces of paper, folded, and placed in the corresponding boxes. Simple random sampling was used to select one county referral hospital from each region. The following hospitals were selected: National and private referral hospitals: Kenyatta National Hospital, Moi Teaching and Referral Hospital, Kisii County Referral Hospital, Jaramogi Oginga Odinga Teaching and Referral Hospital, Nairobi Women Hospitals and Getrudes Children’s Hospital. County Hospitals: Thika County Hospital, Coast General Teaching and Referral Hospital, Marsabit County Hospital, Mbagathi County Referral Hospital, Garissa County Hospital, Nakuru Provincial General Hospital, Homa Bay County Hospital and Kakamega County Hospital. Lastly, collaboration with hospital nursing leadership, nurse managers, ward in-charges, and individual nurses across the selected hospitals was instrumental in facilitating the sharing of the online data collection instrument through hospital-specific WhatsApp groups, direct sharing through email and other platforms.

### Inclusion and exclusion criteria

This study included all nurses, (1) holding valid practice licences from the Nursing Council of Kenya, (2) who consented to this study and (3) who had been employed at the selected hospitals for a minimum of 12 consecutive months. Nurses who did not consent were excluded from the study. Additionally, responses from nurses with less than one year of continuous service were excluded from the analysis.

### Data collection

The data collection was conducted between October 23rd, 2024, and February 28th, 2025, in two phases. The first phase took place during the 66th National Nurses Association of Kenya (NNAK) annual scientific conference and Annual General Meeting (AGM) held at Kenyatta University Amphitheatre (October 23rd to 25th,2024), bringing together over 1000 nurses nationwide. This event facilitated critical networking and collaboration with nurses from selected hospitals. The second phase entailed comprehensive data collection follow-up and snowball sampling of nurses from the target hospitals. The second phase involved targeted follow-ups and on-site visits to selected hospitals for cohesive data collection. An online questionnaire was administered, with a secure link and clear instructions shared with consented nurses. To maximise participation, snowball sampling was encouraged, allowing nurses to distribute the link to colleagues, and expanding the reach and diversity of responses. In addition to the online questionnaire, a paper-pencil questionnaire was also administered to the nurses who wished to respond in this format.

### Data collection instruments and measurements

#### Organizational culture

To assess organizational culture, the study used the Kenya adaption of the Practice Environment Scale of the Nursing Work Index (PES-NWI), a validated 30-item questionnaire divided into seven sub-scales [57, 58]. PES-NWI is a tool first created by Lake [58] to measure the organizational culture of nursing work environments. It was first used in 14 top-performing “Magnet” hospitals in the U.S to identify what makes them excel in nursing care at a time the country was facing extreme nurse turnover. Thereafter, it has been validated across several countries including for this study [57, 59] and found valid and reliable. The seven sub-scales include nurse involvement in hospital affairs (3 items), nurses’ professional development and opportunities (4 items), nursing foundation for quality of care (7 items), administrative support and leadership (4 items), nurse manager ability leadership and support of nursing (5 items), staffing and resource adequacy (4 items), and collegial nurse-physician relationship (3 items). Participants rated their responses on a 4-point Likert scale ranging from 1(strongly disagree) to 4(strongly agree). To determine the rating of organizational culture, an average score was calculated for each item, sub-scale and the overall scale. A score above 2.5 indicated a positive(magnet) organizational culture, characterized by its strong ability to attract and retain nurses. In contrast, a score below 2.5 reflects a negative(non-magnet) culture, lacking the essential attributes of a positive organizational culture.

#### Work-related stress

The Nurse Stress Scale (NSS-34 + 2), a modified version of the original NSS-34, was used to assess WRS, incorporating an additional sub-scale on discrimination with two new items. This validated 34-item tool, derived from the original 57-item NSS [60], has been translated and validated in multiple languages and demonstrates high reliability [60–64]. It measures nursing stress across seven sub-scales including *managing death and dying* (7 items), *conflict with physicians* (5 items), *inadequate preparation* (3 items), *lack of support* (3 items), *conflict with other nurses* (5 items), *workload* (6 items), *uncertainty about treatment* (5 items), and *discrimination* (2 items). Responses are scored using a 4-point Likert scale (1-Never, 2 = occasionally, 3 = frequently, and 4 = very frequently). To find the level of stress average scores were calculated for individual items, sub-scale and the overall scale, with stress levels classified as *low stress* (Mean Score < 2.0), *moderate stress* (mean score between 2.01 and 3.0) and *high stress* (> 3.0.).

### Job turnover intention, professional turnover intention and job satisfaction

Job profession turnover intentions, and job satisfaction (JS) were assessed using modified items from previous studies [65, 66]. Use of a single-item measure to assess overall parameters like job or profession turnover intentions, and job satisfaction has been well supported in literature with several studies demonstrating that a single-item measure is both valid and reliable to assess job satisfaction [67–69]. Job turnover intention (TI) was evaluated using two items (1) the likelihood of leaving one’s current hospital in the next five years and (2) the likelihood of seeking employment abroad within the next five years. ProfTI was assessed also using two questions (1) the likelihood of leaving the nursing profession for a non-nursing job in the next 5 years and (2) the likelihood of leaving the nursing profession all together without changing to another profession in the next 5 years. Both were measured on a 4-point Likert scale (1-Very unlikely, 2 = unlikely, 3 = Likely, and 4 = very likely). Scores were averaged and those between 1.0 and 2.49 indicated no TI, while 2.5 to 4.0 signified TI. JS was assessed using two items: (1) overall satisfaction with all aspects of the current nursing job, including OC in the current hospital and (2) the likelihood of recommending the current workplace to others. Responses were measured on a 4-point Likert scale (1 = Very unsatisfied,2 = unsatisfied,3 = satisfied and 4 = very satisfied). Scores were averaged and between 1.0 and 2.49 were classified as unsatisfied while scores between 2.5 and 4.0 were classified as satisfied.

### Instrument validation and pre-testing

The PES-NWI and NSS are highly validated instruments widely used in nursing research. Both tools have undergone extensive validation across different settings and populations including our validation in Kenya for PES-NWI [57] all showing high validity and reliability: overall Cronbach’s alpha was 0.941 with sub-scale reliabilities ranging from 0.722 to 0.865; sampling adequacy was high (KMO = 0.915) with a significant Bartlett’s Test (χ² = 3463.270, df = 465, *p* < .001), and the model demonstrated good fit (GFI = 0.821, CFI = 0.896, NFI = 0.800, RMSEA = 0.063.To ensure contextual relevance and clarity, we conducted pre-testing of the questionnaire using 40 sample questionnaires at Kenyatta University Teaching and Referral Hospital. Feedback from the pre-test was used only to refine and improve the clarity before we rolled out the main data collection.

### Data management and analysis

Data collected through the online Google Form was downloaded in Excel format, cleaned, and then imported into Statistical Package for the Social Sciences (SPSS) version 28 for analysis. Further manual data was added, and data analysis was performed. The reliability of the primary instruments (PES-NWI and NSS) was first evaluated using Cronbach’s alpha. Descriptive statistics, including frequencies, percentages, means, and Standard Deviations (SD), were applied to summarize demographic characteristics, stress levels, prevalence of turnover intentions and organizational culture. Chi-square tests, correlation analysis, and logistic regression were used to examine variable associations and relationships. A bivariate logistic regression analysis was used to determine the predictors of turnover intention.

## Results

### Demographic characteristics of the participants

 The study involved a total of 429 (58.3% female and 41.7% male) nurses. The majority of the respondents 63.2% worked in county hospitals while 19.1% and 17.7% worked in national referral hospitals and private hospitals respectively. Most participants were married (66.4%), while 31.0% were single, and a small percentage (2.6%) were widowed. Regarding age, the participants’ mean age was 34.05 ± 9.07 years. The largest age group distribution was 21–30 years old (41.7%), followed by those aged 31–40 years (38.7%). The older age group above 40 years was 19.6%. In terms of work experience, the vast majority (81.1%) had work experience between 1 and 15 years while only 18.9% had over 16 years of work experience. Religious affiliation was predominantly Christian (95.8%), with a small number of Muslims (3%) and others (1.2%). Regarding education level, the majority held degree in nursing (53.4%), followed by diploma in nursing (33.3%). A smaller portion had certificate in nursing (1.6), master’s degree (11%) and a PhD (0.7). The employment type was mainly permanent and pensionable (59.2%) while 40.8% were on fixed-term contracts. Respondents worked in various hospital departments with 73.4% being general staff, while 26.6% held leadership positions. The work departments varied, with the highest proportion in maternity/gynaecology/postnatal wards (23.3%), followed by outpatient departments (20.5%). Other wards, including medical (13.3%), surgical (4.7%), ICU/theatre and renal (4.9%), and paediatric/newborn units (4.4%). A significant portion (28.9%) worked in other departments. Regarding monthly income, the distribution varied significantly. While 14% earned Ksh.20,000(approx.160 US$), the largest portion (24.5%) earned above Ksh.100,00(Approx.800 US$) followed closely by those earning between ksh.60,001–80,000(approx.470–620 US$) (See Table  1 ).

**Table 1:** Demographic characteristics of the respondents (*n* = 429)

Variables		Frequency n (%)
Gender		179(41.7)
	Female	250(58.3)
	Married	285(66.4)
	Single	133(31.0)
	Widow/Widower	11(2.6)
Age	21–30	179(41.7)
	31–40	166(38.7)
	41–50	51(11.9)
	51–60	33(7.7)
Years of Experience	≤ 5 yrs	194(45.2)
	6-15yrs	154(35.9)
	16-25yrs	45(10.5)
	≥ 26yrs	36(8.4)
Religion	Christian	411(95.8)
	Muslim	13(3.0)
	Others	5(1.2)
Highest Education	Certificate in Nursing	7(1.6)
	Diploma in Nursing	143(33.3)
	BScN	229(53.4)
	Masters	47(11.0)
	PhD	3(0.7)
Type of hospital	County hospital	271(63.2)
	National hospital	82(19.1)
	Private hospitals	76(17.7)
Designations	General nursing staff	315(73.4)
	Nursing staff with leadership position	114(26.6)
Ward	Outpatient department (including outpatient special clinics)	88(20.5)
	Maternity ward/Gynecology ward/postnatal ward	100(23.3)
	Medical wards (Male/Female)	57(13.3)
	Surgical wards (Male/Female)	20(4.7)
	Intensive Care Unit/Renal unit/Theatre"	20(4.9)
	Paediatrics ward/ Newborn Unit	19(4.4)
	Others	129(28.9)
Type of employment	Permanent & pensionable	254(59.2)
	Fixed term Contracts	175(40.8)
Monthly income	Below KSh 20,000	60(14.0)
	KSh 20,001–40,000	74(17.2)
	KSh 40,001–60,000	28(6.5)
	KSh 60,001–80,000	104(24.2)
	KSh 80,001–100,000	59(13.5)
	Above Ksh 100,000	104(24.5)

### Objective I: characterizing the prevailing organizational culture in kenya’s major referral hospitals as viewed by nurses

Our research measured organization culture among Kenyan nurses using the Kenyan version of PES-NWI-30 which demonstrated high reliability in this study Cronbach’s alpha (0.938). The overall organizational culture rating was 2.54(SD = 0.62), indicating that the OC was largely neutral with a slight inclination to positive culture. Among the seven sub-scales, five; nurses involvement in hospital affairs (M = 2.54, SD = 0.90), nursing foundation for quality of care(M = 2.57, SD = 0.75), administrative support and leadership(M = 3.13, SD = 0.70), nurse manager ability leadership and support of nursing(M = 2.59, SD = 0.79) and collegial nurse-physician relationships (M = 2.69, SD = 0.85) were found to be positive. However, two sub-scales, nurses’ professional development and opportunities (M = 2.39, SD = 0.82) and staffing and resources adequacy (M = 2.15, SD = 0.88) (See Table 2).

**Table 2:** Rating of kenya’s organizational culture by Kenyan nurses *n* = 429

Sn	Sub-Scale	No of Items	Mean (SD)	Classification
1.	Nurse Involvement in Hospital Affairs (OC1)	3	2.54(0.90)	Positive
2.	Nurses’ Professional development and opportunities (OC2)	4	2.39(0.82)	Negative
3.	Nursing Foundation for quality of care (OC3)	7	2.57(0.75)	Positive
4.	Administrative Support and Leadership (OC4)	4	3.13(0.70)	Positive
5.	Nurse Manager Ability, Leadership, and Support of Nursing (OC5)	5	2.59(0.79)	Positive
6.	Staffing and Resource Adequacy (OC6)	4	2.15(0.88)	Negative
7.	Collegial Nurse–Physician Relations (OC7)	3	2.69(0.85)	Positive
**Overall rating of the organizational culture**		**30**	**2.54(0.62)**	**Positive**

### Objective II: the level of work-related stress among nurses working in kenya’s major referral hospitals

Work-related stress was assessed using the 36-item Nurses Stress Scale (NSS), which showed excellent reliability (Cronbach’s α = 0.920). The overall findings indicated that the level of WRS among Kenyan nurses was moderately high (M = 2.92, SD = 0.51), with three sub-scale domains exhibiting high-stress levels: handling death and dying (M = 3.24, SD = 0.49), high workloads (M = 3.20, SD = 0.57), and discrimination (M = 3.10, SD = 0.97) (Table 3). Other sub-scales, including conflict with physicians(M = 2.81), inadequate preparation(M = 2.69), lack of support(M = 2.66), conflict with other nurses(M = 2.68), and uncertainty concerning treatment(M = 3.00), indicated moderate levels of work-related stress.

**Table 3:** Levels of work-related stress among Kenyan nurses *n* = 429

Sn	Sub-Scale	No of items	Mean (SD)	Classification
1.	Death and Dying (NSS1)	7	3.24(0.49)	High stress
2.	Conflict with Physicians (NSS2)	5	2.81(0.65)	Moderate stress
3.	Inadequate Preparation (NSS3)	3	2.69(0.76)	Moderate stress
4.	Lack of Support (NSS4)	3	2.66(0.78)	Moderate stress
5.	Conflict with Other Nurses (NSS5)	5	2.68(0.81)	Moderate stress
6.	Workload (NSS6)	6	3.20(0.57)	High stress
7.	Uncertainty Concerning Treatment (NSS7)	5	3.00(0.68)	Moderate stress
8.	Discrimination (NSS8)	2	3.10(0.97)	High stress
**Overall Level of Work-Related Stress Among Kenyan Nurses**		**36**	**2.92(0.51)**	Moderate stress

WRS was classified as **Low** (Mean Score < 2.0) **Moderate** levels of stress (Mean score between 2.01 to 3.0) **High** levels of stress (> 3.0.)

### Objective III&IV: the prevalence of job turnover intention, professional turnover intention and job satisfaction among nurses working in kenya’s major referral hospitals

**Graph 1:**
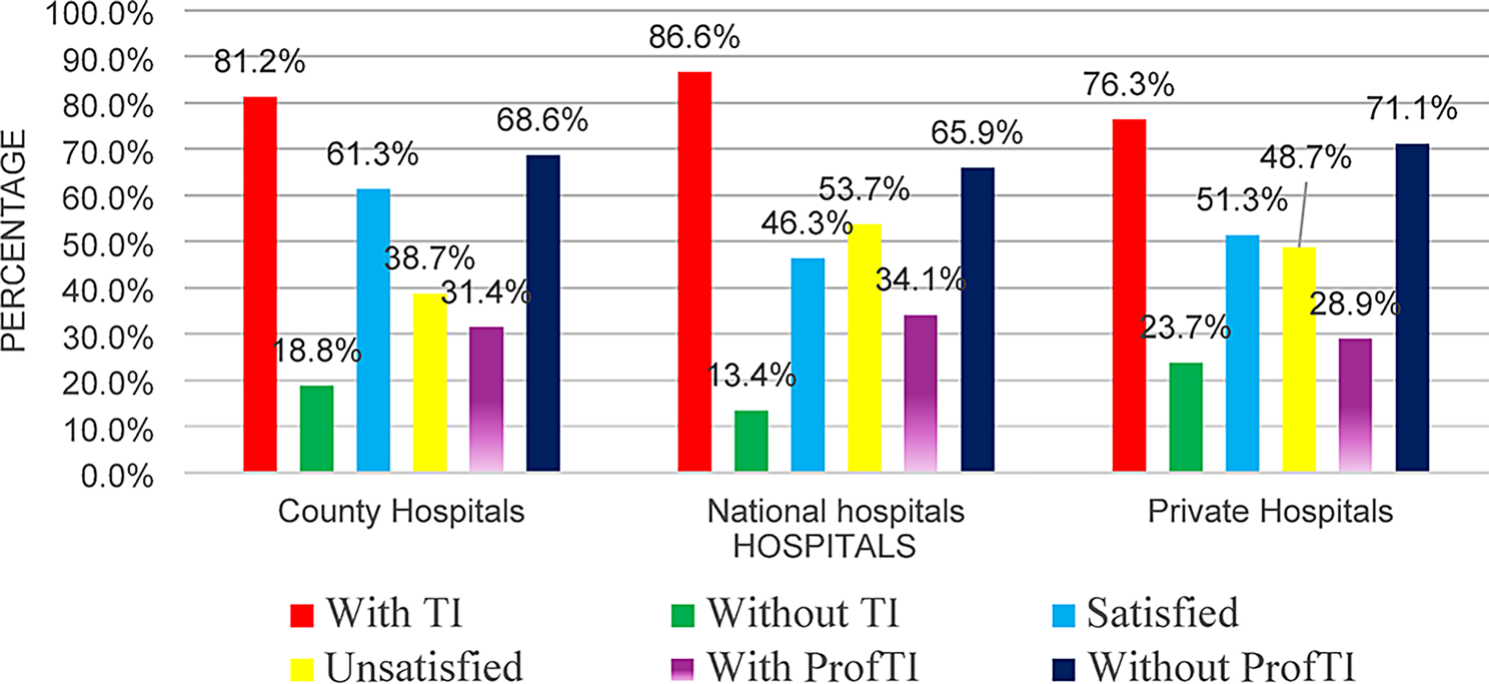
Job TI, Professional TI and job satisfaction among Kenyan nurses

Among the 429 nurses surveyed (271 from county hospitals, 82 from national referral hospitals and 76 from private hospitals), the overall job TI among nurses was found to be high at 81.4%(*n* = 349) signaling a profound workforce crisis in the country. National referral hospitals exhibited the highest attrition risk, with 86.6% of nurses intending to leave, followed by county hospitals (81.2%) and private hospitals (76.3%). The overall ProfTI was found to be 31.5%(*n* = 135) with majority 68.5% (*n* = 294) of the nurses indicating no desire to leave the nursing profession. Job satisfaction remained alarmingly low, with an overall 56.6%(*n* = 243) of nurses across all three hospitals reporting to be satisfied, while 43.6%(*n* = 186) were dissatisfied. Job satisfaction differed across the three hospital types, with 61.3% of the nurses in county hospitals, 51.3% in private hospitals and 46.3% in national referral hospitals reporting being satisfied (Graph  1).

### Objective V: predicting the job turnover intention and professional turnover intention among nurses working in kenya’s major referral hospitals using organizational culture, work-related stress and job satisfaction

#### Chi-square results

A chi-square test was conducted to examine the association between various variables with TI and ProfTI. Among demographic variables, age(*P* = .000), years of work experience(*P* = .000), and marital status(*P* = .000) showed significant association with TI while marital status(*P* = .002), age (*P* = .009), highest education level(*P* = .007), designation (*P* = .001), ward(*P* = .001), monthly income/salary(*P* = .004) significantly associated with ProfTI see Supplementary Table 1. Additionally, all sub-scales of organizational culture (OC1-OC7), work-related stress (NSS1-NSS8) and job satisfaction demonstrated a strong association with both TI and ProfTI (*P* < 001), reinforcing their critical role in shaping nurses’ TI and ProfTI.

#### Pearson correlations results

First, we performed a correlation analysis to explore the relationship between TI, ProfTI, JS and sub-scales of organizational culture (OC1-OC7) and WRS(NSS1-NSS8) (Table 4).

##### Correlation between intention to leave nursing career and job satisfaction

- TI vs. JS (*R*=-.153, P = < 001).
- ProfTI vs.JS (-0.207, P = < 001).
- A moderate negative correlation suggests that higher job satisfaction lowers both TI and ProfTI.

##### Correlation between organizational culture and intention to leave career

- Although not statistically significant, five OC sub-scales; *nurse involvement in hospital affairs* (OC1), *nurses’ professional development and opportunities* (OC2), the *nursing foundation for quality of care* (OC3), *administrative support and leadership* (OC4) and *nurse manager ability*,* leadership*,* and support of nursing* (OC5), all had negative correlation with TI but not ProfTI. Sub-scales: *staffing and resource adequacy* (OC6) and *collegial nurse-physician relations* (OC7) had positive but non-significant correlations.
- This suggests that positive OC(OC1-OC5) indirectly reduce TI while negative OC (OC6 & OC7) indirectly increase TI.
- OC and ProfTI had non-significant correlations.
- Notably, various OC dimensions (OC1-OC7) and Job satisfaction showed a significant positive correlation ((e.g. OC1, *R* = .133, P = < 001), (OC2, *R* = .175, P = < 001) and (OC3, *R* = .102, P = < 001)) suggesting that strong positive OC enhanced job satisfaction and retention.

##### Correlations between work-related stress, job and professional turnover intentions

Contrary to expectations, almost all WRS sub-scales (NSS1-NSS8), negatively correlated with TI, with several sub-scales (NSS2(*R*=-.204, P = < 001) and Inadequate Preparation-NSS3(*R*=-.154, P = < 001) showing statistically significant results. Conversely, ProfTI significantly and positively related with WRS with almost all the sub-scales showing positively significant correlations with ProfTI.

##### Work-related stress and job satisfaction- A complex relationship

While overall work-related stress negatively correlated with job satisfaction, specific stress sub-scales NSS2(*R* = .192, P = < 001) and NSS4(*R* = .136, P = < 001) showed a significantly positive correlation suggesting that some nurses may endure stress yet still report moderate satisfaction.

**Table 4:** Correlations between intention to leave career, job satisfaction and sub-scales of organizational culture and work-related stress *n* = 429

Correlations																		
	1.	2.	3.	4.	5.	6.	7.	8.	9.	10.	11.	12.	13.	14.	15.	16.	17.	18.
1. TI	1																	
2. ProfTI	0.144^**^	1																
3. JS	− 0.153^**^	− 0.207^**^	1															
4. OC1	− 0.055	0.071	0.133^**^	1														
5. OC2	− 0.063	− 0.032	0.175^**^	0.599^**^	1													
6. OC3	− 0.040	0.047	0.102^*^	0.488^**^	0.599^**^	1												
7. OC4	− 0.035	0.048	0.091	0.455^**^	0.463^**^	0.572^**^	1											
8. OC5	− 0.039	0.002	0.092	0.563^**^	0.570^**^	0.633^**^	0.640^**^	1										
9. OC6	0.010	0.051	0.033	0.299^**^	0.450^**^	0.499^**^	0.270^**^	0.453^**^	1									
10. OC7	0.053	0.066	− 0.010	0.369^**^	0.405^**^	0.535^**^	0.536^**^	0.578^**^	0.436^**^	1								
11. NSS1	− 0.028	− 0.006	− 0.119^*^	0.008	− 0.036	− 0.011	0.036	− 0.089	− 0.180^**^	− 0.045	1							
12. NSS2	− 0.204^**^	0.044	0.192^**^	0.048	0.055	0.069	− 0.008	0.014	− 0.167^**^	0.019	0.308^**^	1						
13. NSS3	− 0.154^**^	0.085	0.033	0.032	− 0.057	0.095^*^	0.050	0.033	− 0.100^*^	0.131^**^	0.256^**^	0.458^**^	1					
14. NSS4	− 0.087	0.166^**^	0.136^**^	0.040	− 0.041	0.083	0.104^*^	− 0.006	− 0.169^**^	0.138^**^	0.094	0.481^**^	0.543^**^	1				
15. NSS5	− 0.012	0.131^**^	0.154^**^	− 0.034	− 0.153^**^	− 0.064	0.016	− 0.071	− 0.225^**^	0.062	0.212^**^	0.530^**^	0.447^**^	0.690^**^	1			
16. NSS6	− 0.002	0.123^*^	− 0.129^**^	− 0.051	− 0.032	0.030	− 0.041	− 0.075	− 0.095	0.037	0.427^**^	0.572^**^	0.463^**^	0.417^**^	0.340^**^	1		
17. NSS7	− 0.056	0.167^**^	0.128^**^	0.056	0.028	0.046	0.050	0.064	− 0.087	0.091	0.297^**^	0.650^**^	0.514^**^	0.476^**^	0.451^**^	0.612^**^	1	
18. NSS8	− 0.057	− 0.015	0.027	0.038	0.047	0.110^*^	0.016	0.072	− 0.048	0.065	0.057	0.482^**^	0.449^**^	0.437^**^	0.267^**^	0.526^**^	0.687^**^	1

**. Correlation is significant at the 0.01 level (2-tailed)

*. Correlation is significant at the 0.05 level (2-tailed)

**KEY: TI-**Job Intention to Leave Career, P**rofTI-**Profession turnover Intention, **JS-**Job Satisfaction, **OC-**Organizational culture, **NSS**-Nursing Stress Scale

#### Regression analysis: predictors of job and professional turnover intention among nurses working in major referral hospitals in Kenya

A binary logistic regression was conducted to determine significant predictors of nurses’ TI and ProfI (Tables 4 and 5). Prior to regression analysis, multicollinearity was assessed using Variance Inflation Factors (VIFs), and all predictors had VIF values ​​below 5, indicating no significant multicollinearity.

### Demographic predictors of job and professional turnover intention among the nurses

For job turnover intentions, nurses aged 21–40 were significantly less likely to leave their current jobs compared to those aged 41–60(OR = 0.045.95% CI [0.26,0.078], *P* = .004). Additionally, nurses with 5 years or less work experience were significantly less likely to intend to quit their current job compared with those with 26 years or more years of experience (OR = 0.40, 95% CI [0.17, 0.93], *P* = .033) Table 4. Regarding profession turnover intention, married nurses were 3 times more likely to intend to leave the profession than single nurses (OR = 3.19, 95% CI [1.70,5.99], *P* = .001). Nurses were also found to be 11 to 13 times more likely to leave the profession if they were working in surgical wards (OR = 12.70, 95% CL [1.48,108.85], *P* = .020) or in ICU/renal/theatre (OR = 10.79, 95% CI [1.27, 91.45] *P* = .029) compared to work in other wards. Furthermore, nurses earning lower salaries between Ksh.20,000–40,000 (OR = 2.55, 95% CI[1.03, 6.29] *P* = .042) and 40,001–60,000(OR = 4.91,95% CI[1.21,19.92], *P* = .026) —translating to salary less than 500USD) were three to five times likely to leave the nursing profession compared to those earning above Ksh100,000(800USD). Conversely, those earning between Ksh.80,001-100,000 were less likely to report intention to leave the profession (OR = 0.43, 95% CI [0.20, 0.93], *P* = .33) Table 5.

**Table 5:** Logistic regression of demographic predictors of job and professional turnover intention among nurses in Kenya

Dependent Variables	Independent Variables		B	Std. Error	Wald	df	Sig.	Exp(B) OR	95% Confidence Interval for Exp(B)	
									Lower Bound	Upper Bound
**TI**	Marital Status	Married	0.157	0.327	0.230	1	0.632	1.170	0.617	2.219
		Singles	Ref			0				
	Age group	21–40	− 0.804	0.280	8.224	1	**0.004**	0.448	0.258	0.775
		41–60	Ref			0				
	Work experience	≤ 5 yrs	− 0.910	0.428	4.532	1	**0.033**	0.402	0.174	0.930
		6-15yrs	− 0.638	0.430	2.206	1	0.137	0.528	0.227	1.226
		16-25yrs	0.550	0.481	1.309	1	0.252	1.733	0.676	4.447
		≥ 26yrs	Ref			0				
**ProfTI**	Marital Status	Married	1.161	0.321	13.122	1	**0.000**	3.193	1.704	5.985
		Singles	Ref			0				
	Age group	21–40	0.304	0.255	1.425	1	0.233	1.355	0.823	2.233
		41–60	Ref							
	Highest Education level	Certificate & Diploma	0.442	0.339	1.696	1	0.193	1.556	0.800	3.024
		BScN	0.415	0.322	1.657	1	0.198	1.514	0.805	2.848
		Masters & PhD	Ref							
	Designation	General nursing staff	0.395	0.318	1.545	1	0.214	1.484	0.796	2.766
		Nursing staff with leadership position	Ref			0				
	Ward	Outpatient department (including outpatient special clinics)	− 0.027	0.375	0.005	1	0.944	0.974	0.467	2.029
		Maternity ward/Gynecology ward/postnatal ward	− 0.491	0.363	1.829	1	0.176	0.612	0.300	1.247
		Medical wards (Male/Female)	0.324	0.406	0.634	1	0.426	1.382	0.623	3.066
		Surgical wards (Male/Female)	2.541	1.096	5.376	1	**0.020**	12.699	1.482	108.845
		Intensive Care Unit/Renal unit/Theatre”	2.378	1.091	4.755	1	**0.029**	10.786	1.272	91.454
		Paediatrics ward/ Newborn Unit	− 0.360	0.605	0.354	1	0.552	0.698	0.213	2.283
		Others	Ref			0				
	Monthly income/Salary	Below KSh 20,000	− 0.266	0.418	0.405	1	0.524	0.766	0.338	1.739
		KSh 20,001–40,000	0.936	0.460	4.130	1	**0.042**	2.549	1.034	6.286
		KSh 40,001–60,000	1.591	0.715	4.955	1	**0.026**	4.908	1.209	19.921
		KSh 60,001–80,000	0.321	0.400	0.645	1	0.422	1.379	0.630	3.021
		KSh 80,001–100,000	− 0.851	0.399	4.559	1	**0.033**	0.427	0.195	0.933
		Above Ksh 100,000	Ref			0				

**TI** = Job Turnover Intention, **ProfTI** = Profession Turnover Intention

### Organizational culture, work-related stress and job satisfaction as predictors of job and professional turnover intention among the nurses

Analysis revealed that job satisfaction significantly reduced both job and professional turnover intentions among nurses. Higher levels of job satisfaction were associated with lower odds of TI (OR = 0.45,95% CI [0.26, 0.77], *P* = .004) and ProfTI(OR = 35, 95% CI [0.23, 0.54], P = < 0.001). Contrary to most existing literature, the study uncovered that work-related stress is inversely related to TI (OR = 0.63, 95% CI [0.40, 0.99], *P* = .046), but positively associated with ProfTI(OR = 1.74, 95%, CI [1.15. 2.61], *P* = .008). Organizational culture was not a significant predictor of either job (OR = 1.02, *P* = .942) or professional turnover intention (OR = 1.51, *P* = .060) Table 6.

**Table 6:** Binary logistic regression on job turnover intention, profession turnover intention, organizational culture job satisfaction, and Work-related stress

Dependent Variable	Independent Variables	B	S.E.	Wald	Df	Sig.	(Exp(B) OR	95% C.I.for EXP(B)		Major Effects on TI, ProfTI & Conclusion
								Lower	Upper	
**TI**	OC (OC1-OC7)	0.019	0.255	0.005	1	0.942	1.019	0.618	1.679	No direct significant effects of OC on TI
	JS	− 0.800	0.277	8.330	1	**0.004**	0.449	0.261	0.774	Lower job satisfaction increased TI
	NSS (NSS1-8)	− 0.465	0.233	3.976	1	**0.046**	0.628	0.397	0.992	High job stress reduced TI
	Constant	3.898	0.803	23.577	1	0.000	49.288	-	-	-
**ProfTI**	OC (OC1-OC7)	0.413	0.220	3.531	1	0.060	1.512	0.982	2.327	No direct significant effects of OC on ProfTI
	JS	-1.055	0.223	22.410	1	**0.000**	0.348	0.225	0.539	JS significantly reduce ProfTI
	NSS (NSS1-8)	0.552	0.208	7.015	1	**0.008**	1.736	1.154	2.611	High levels of WRS increase ProfTI
	Constant	-1.143	0.649	3.106	1	0.078	0.319	-	-	-

**TI** = Job Turnover Intention, **ProfTI** = Profession Turnover Intention

## Discussion

Understanding the key drivers of nurses’ job and professional turnover intentions in LMICs, especially in Kenya, where nurse exodus is high, is vital for developing actionable retention strategies. To the best of our knowledge, this is the first study to comprehensively explore the effects of organizational culture, work-related stress and job satisfaction on nurses’ TI and ProfTI in Kenya.

This study revealed that the overall organizational culture in Kenya’s major referral hospitals was largely neutral with a slight inclination towards positive. Work-related stress was moderately high, while job satisfaction was low, with only 56.6% of respondents across the major referral hospitals reporting satisfaction. Furthermore, it revealed that Kenya’s major referral hospitals face a severe crisis, with 81.4% of nurses expressing intent to leave their jobs—highest in government-owned facilities; national referral hospitals (86.6%), followed by county referral hospitals (81.2%) while lower in private referral hospitals (76.3%) respectively. Although most nurses (68.5%) intend to stay in the profession, a troubling one-third (31.5%) consider leaving the profession entirely is a point of concern. These findings are consistent with those reported in other studies across LMICs, including research conducted in Ghana [70], SSA [1], and Ethiopia [71].

This study uncovered critical demographic factors driving jobs and professional turnover intentions in the referral hospitals. Younger nurses (21–40 years) and those with less than five years of work experience were significantly less likely to intend to leave their current jobs, in contrast with their older, more experienced counterparts. This trend may reflect a generational difference in job expectations or resilience and highlight the potential retention value of early-career nurses if adequately supported. However, the high turnover intention among nurses 41–60 years and with 26 + years of experience signals a looming loss of highly experienced experts, demanding urgent action through phased retirement or job enrichment strategies. Furthermore, professional turnover intention data unveils alarming vulnerabilities: married nurses were over three times more likely to express intent to exit the profession, possibly due to compounded pressure of work-family conflict and demanding nature of nursing shifts in high-acuity areas such as surgical, and ICU/renal/theatre wards. The staggering odds ratios for ProfTI in these specialized units ranging from 10 to 13 times higher than in other wards underscore emotional, physical and psychological toll exacted by such roles, likely exacerbated by chronic stress and burnout something many studies have highlighted [72–74]. Salary is a major predictor of TI and ProfTI [11, 70]. This study found that nurses earning less than 500USD were significantly more likely to intend to quit the profession, suggesting that the current salary scale in many referral facilities in Kenya are failing to meet the economic and motivational threshold for retention.

Contrary to expectations and prior literature that often position organizational culture as a key determinant of TI and ProfTI [29, 30, 34, 36, 38, 39, 41], our analysis revealed that organizational culture did not significantly predict both TI and ProfTI. This suggests that the largely neutral OC that was exhibited by Kenya’s referral hospitals, was too diffuse or weakly internalized to exert measurable influence on career and professional decisions.

In a striking departure from existing literature, this study found that work-related stress was a significant negative predictor of TI (B=-0.465, *P* = .046, OR = 0.628), suggesting that even highly stressed nurses were less likely to consider quitting their jobs. These findings contradict a vast body of research which have found that elevated work-related stress is a major driver of TI and actual attrition in nursing [30, 48, 49, 75]. However, in the Kenyan context, this inverse relationship may be attributed to prolonged nurses’ exposure to work-related stress, which cultivates resilience, perseverance, endurance, hardiness and adaptability among nurses [76]. Additionally, factors such as limited alternative job options, the esteemed status of the nursing profession in Kenya, high unemployment, and difficult immigration processes may further deter job abandonment. This complex phenomenon warrants deeper investigation to fully understand. However, consistent with other studies, we found that WRS positively and significantly elevated ProfTI, indicating that while nurses may tolerate stress in the short term, sustained stress over time may push them to consider leaving the profession entirely.

Furthermore, this study found that job satisfaction was the most powerful predictor of both TI and ProfTI. The analysis revealed that nurses with higher job satisfaction were less likely to consider leaving their current jobs (OR = 0.45, 95% CI [0.26, 0.77], *P* = .004) or exiting the nursing profession entirely (OR = 0.35, 95% CI [0.23, 0.54], *P* < .001). Defined as the extent to which nurses feel happy, content, and positive about their nursing jobs and profession [77–79], job satisfaction has widely been recognized as a key factor reducing both TI and nurse ProfTI [29, 35, 65, 80]. Consistent with previous studies, our findings indicate that nurses who experience higher satisfaction are significantly more likely to remain in their positions and the profession. Job satisfaction not only mitigates work-related stress but also enhances nurses’ mental, emotional and physical well-being. Moreover, studies have found that satisfied nurses demonstrate greater efficiency, productivity and professional and career commitment factors that are catalysts for improved healthcare service delivery and a better patient outcome. The strong inverse relationship between job satisfaction, work-related stress and TI underscores the urgent need for healthcare institutions in Kenya to prioritize and implement strategies that enhance job satisfaction. Key measures should include fostering a strong positive organizational culture, improving work environments, ensuring fair compensation, and providing career and professional development opportunities.

### Study’s implication for policy and practice

The study’s findings have significant implications for healthcare policy and practice in Kenya and other LMICs. First, the high levels of intention to leave both jobs and the nursing profession, low levels of job satisfaction, weak organizational culture and high levels of work-related stress signal an urgent need for systemic reforms. Healthcare institutions must prioritize and enhance job satisfaction. Job satisfaction is like a moving target, ever-evolving and changing—what fulfils and motivates today may not hold the same value tomorrow. However, building a strong positive organizational culture characterized by respect for nurses, teamwork, collaboration, nurses’ involvement in leadership and decision-making, effective communication, flexible work schedules, adequate resources and staffing, increasing opportunities for career and academic progression, adequate pay, regular recognition, good leadership styles, and with decent work environments, and any other factor increasing job satisfaction should be prioritized. Secondly, targeted interventions to reduce work-related stress such as psychological support, mentorship programs, swift conflict resolutions, and reducing discrimination could help alleviate work-related stress and reduce TI and ProfTI. These programs should also be tailored to meet individual nurses demographics such as age, marital status, department/ward among other specifications.

## Conclusion

This study provides critical insights into crisis in Kenya’s major referral hospitals, the country’s topmost health facilities, revealing that only 56.6% of the country’s nurses were satisfied with their jobs, and an alarmingly 81.4% and 31.5% of nurses in the country intended to exit jobs and the nursing profession respectively. Moreover, the country’s organizational culture was found largely neutral, offering limited influence on nurses’ decisions to stay or leave. Job satisfaction emerged as the most powerful predictor of TI and ProfTI, significantly reducing the likelihood of wanting to leave. While WRS was moderately high, the study uncovered surprisingly contradictory inverse relationship between TI and WRS. This suggests that stress may build resilience, hardiness, perseverance, and a strong sense of professional commitment, rather than prompting nurses to leave job. However, WRS drove ProfTI indicating limits to stress endurance. These findings demand urgent strategic interventions. Nurse managers, hospital leaders, policymakers, the Kenyan government and all other stakeholders must prioritize enhancing job satisfaction, cultivating a strong positive organizational culture, mitigating WRS to stem the tide of nurse job and professional turnover intentions from leading to real turnover. This will safeguard healthcare systems.

### Limitations and future research direction

The findings of this study should be interpreted with caution due to several limitations. First, the cross-sectional study design limits causal inferences, making it difficult to establish definitive cause-and-effect relationships between study variables. Self-reported measures may introduce bias. Secondly, the use of self-reported measuring instruments for data collection may introduce social desirability and recall bias, potentially affecting the accuracy of the responses. Future research should consider longitudinal study designs to track changes in turnover and turnover intentions over time and better capture the evolving nature of organizational culture, job satisfaction and work-related stress. Moreover, qualitative or mixed-methods approaches could provide richer insights. Further exploration of individual coping mechanisms, resilience, and adaptation strategies could enhance our understanding of the interplay between work-related stress and nurses’ retention offering more nuanced policy interventions and recommendations.

## Electronic supplementary material

Below is the link to the electronic supplementary material.

Supplementary Material 1

## Data Availability

All data generated and analysed during this study are included in the manuscript and supplementary files. Additional data can be obtained from the corresponding author upon reasonable request.

## Abbreviations

AGM: Annual General Meeting
BScN: Bachelor of Science in Nursing
ICU: Intensive Care Unit
ITProf: Intention to Leave Profession
JS: Job Satisfaction
Ksh: Kenya Shilling
LMICs: Low- and Middle-Income Countries
M: Mean
NACOSTI: National Commission for Science, Technology and Innovation
NCK: Nursing Council of Kenya
NNAK: National Nurses Association of Kenya
NSS: Nursing Stress Scale
OC: Organizational Culture
OR: Odds Ratio
PES-NWI: Practice Environment Scale of Nursing Work Index
PhD: Doctor of Philosophy
ProfTI: Profession Turnover Intention
SD: Standard Deviation
TI: Job Turnover Intention
US$: United States Dollar
WHO: World Health Organization
WRS: Work-Related Stress
