# Supplementary material for: Intentions to leave the job and nursing profession among nurses in Kenya’s referral hospitals: exploring the effects of organizational culture, work-related stress and job satisfaction
Source: BMC Nurs. 2025 Jul 2;24:823. doi: 10.1186/s12912-025-03422-0 (PMC12220216; doi:10.1186/s12912-025-03422-0)
Supplement: Supplementary file 1 — Supplementary Material 1 [file 12912_2025_3422_MOESM1_ESM.docx]

**SUPPLIMENTARY Table 1:Chi-Square ASSOCIATION BETWEEN DEMOGRAPHIC VARIABLES WITH TI and ProfTI**

| **Chi-Square ASSOCIATION BETWEEN DEMOGRAPHICS AND TI** | | | | | | |
| --- | --- | --- | --- | --- | --- | --- |
| **Variable** | **Category** | **ITLc** | **No ITLc** | **Chi-Square(χ2)** | **DF** | **P-Value** |
| **Gender** | Male | 149 | 30 | .722 | 1 | .396 |
|  | Female | 200 | 50 |  |  |  |
| **Marital Status** | Married | 228 | 57 | 30.681 | 3 | **.000** |
|  | Single | 116 | 17 |  |  |  |
|  | Widow/Widower | 5 | 8 |  |  |  |
| **Age** | 21-30 | 156 | 23 | 16.595 | 3 | **.001** |
|  | 31-40 | 134 | 32 |  |  |  |
|  | 41-50 | 40 | 11 |  |  |  |
|  | 51-60 | 19 | 14 |  |  |  |
| **Years of Experience** | ≤5 yrs | 168 | 26 | 19.337 | 3 | **.000** |
|  | 6-15yrs | 128 | 26 |  |  |  |
|  | 16-25yrs | 27 | 18 |  |  |  |
|  | ≥26yrs | 26 | 10 |  |  |  |
| **Religion** | Christian | 332 | 79 | 2.264 | 2 | .322 |
|  | Muslim | 12 | 1 |  |  |  |
|  | Others | 5 | 0 |  |  |  |
| **Highest Education** | Certificate in Nursing | 5 | 2 | 4.276 | 5 | .510 |
|  | Diploma in Nursing | 118 | 25 |  |  |  |
|  | BScN | 186 | 43 |  |  |  |
|  | Masters | 39 | 10 |  |  |  |
|  | PhD | 1 | 0 |  |  |  |
| **Designations** | General nursing staff | 262 | 53 | 2.596 | 1 | .107 |
|  | Nursing staff with leadership position | 87 | 27 |  |  |  |
| **Ward** | Outpatient department (including outpatient special clinics) | 73 | 15 | 7.013 | 6 | .320 |
|  | Maternity ward/Gynecology ward/postnatal ward | 75 | 25 |  |  |  |
|  | Medical wards (Male/Female) | 48 | 9 |  |  |  |
|  | Surgical wards (Male/Female) | 19 | 1 |  |  |  |
|  | Intensive Care Unit/Renal unit/Theatre" | 19 | 2 |  |  |  |
|  | Paediatrics ward/ Newborn Unit | 16 | 3 |  |  |  |
|  | Others | 99 | 25 |  |  |  |
| **Type of employment** | Permanent & pensionable | 145 | 30 | .441 | 1 | .506 |
|  | Fixed term Contracts | 204 | 50 |  |  |  |
| **Monthly income** | Below KSh 20,000 | 47 | 13 | 7.721 | 5 | .172 |
|  | KSh 20,001 – 40,000 | 64 | 10 |  |  |  |
|  | KSh 40,001 – 60,000 | 25 | 3 |  |  |  |
|  | KSh 60,001 – 80,000 | 88 | 16 |  |  |  |
|  | KSh 80,001 – 100,000 | 42 | 17 |  |  |  |
|  | Above Ksh 100,000 | 83 | 21 |  |  |  |
| **Chi-Square ASSOCIATION BETWEEN DEMOGRAPHICS AND ProfTI** | | | | | | |
| **Variable** | **Category** | **With**  **ProfTI** | **No**  **ProfTI** | **Chi-Square(χ2)** | **DF** | **P-Value** |
| **Gender** | Male | 58 | 121 | .124 | 1 | .725 |
|  | Female | 77 | 171 |  |  |  |
| **Marital Status** | Married | 77 | 205 | 6.619 | 1 | **.002** |
|  | Single | 57 | 87 |  |  |  |
|  | Widow/Widower | 1 | 2 |  |  |  |
| **Age** | 21-30 | 59 | 120 | 11.634 | 3 | **.009** |
|  | 31-40 | 45 | 121 |  |  |  |
|  | 41-50 | 25 | 26 |  |  |  |
|  | 51-60 | 6 | 27 |  |  |  |
| **Years of Experience** | ≤5 yrs | 60 | 134 | 3.202 | 3 | .362 |
|  | 6-15yrs | 47 | 107 |  |  |  |
|  | 16-25yrs | 19 | 26 |  |  |  |
|  | ≥26yrs | 9 | 27 |  |  |  |
| **Religion** | Christian | 127 | 284 | 5.525 | 2 | .063 |
|  | Muslim | 4 | 9 |  |  |  |
|  | Others | 4 | 1 |  |  |  |
| **Highest Education** | Certificate in Nursing | 0 | 7 | 15.937 | 5 | **0.007** |
|  | Diploma in Nursing | 45 | 98 |  |  |  |
|  | BScN | 70 | 159 |  |  |  |
|  | Masters | 19 | 30 |  |  |  |
|  | PhD | 0 | 1 |  |  |  |
| **Designations** | General nursing staff | 113 | 202 | 10.663 | 1 | **.001** |
|  | Nursing staff with leadership position | 22 | 92 |  |  |  |
| **Ward** | Outpatient department (including outpatient special clinics) | 22 | 66 | 23.759 | 6 | **.001** |
|  | Maternity ward/Gynecology ward/postnatal ward | 38 | 62 |  |  |  |
|  | Medical wards (Male/Female) | 20 | 37 |  |  |  |
|  | Surgical wards (Male/Female) | 1 | 19 |  |  |  |
|  | Intensive Care Unit/Renal unit/Theatre" | 1 | 20 |  |  |  |
|  | Paediatrics ward/ Newborn Unit | 11 | 8 |  |  |  |
|  | Others | 42 | 82 |  |  |  |
| **Type of employment** | Permanent & pensionable | 55 | 120 | .000 | 1 | .988 |
|  | Fixed term Contracts | 80 | 174 |  |  |  |
| **Monthly income** | Below KSh 20,000 | 23 | 37 | 17.273 | 5 | **.004** |
|  | KSh 20,001 – 40,000 | 21 | 53 |  |  |  |
|  | KSh 40,001 – 60,000 | 3 | 25 |  |  |  |
|  | KSh 60,001 – 80,000 | 27 | 77 |  |  |  |
|  | KSh 80,001 – 100,000 | 29 | 30 |  |  |  |
|  | Above Ksh 100,000 | 32 | 72 |  |  |  |
